# The Effect of Reduced Dietary Protein on Adipose Tissue in Local Krškopolje Pigs

**DOI:** 10.3390/ijms26094440

**Published:** 2025-05-07

**Authors:** Klavdija Poklukar, Marjeta Čandek-Potokar, Milka Vrecl, Jana Brankovič, Matjaž Uršič, Martin Škrlep

**Affiliations:** 1 Agricultural Institute of Slovenia, Hacquetova 17, 1000 Ljubljana, Slovenia; klavdija.poklukar@kis.si (K.P.); meta.candek-potokar@kis.si (M.Č.-P.); 2 Institute of Preclinical Sciences, Veterinary Faculty, University of Ljubljana, Gerbičeva ulica 60, 1000 Ljubljana, Slovenia; milka.vrecl@vf.uni-lj.si (M.V.); jana.brankovic@vf.uni-lj.si (J.B.);

**Keywords:** pig, local breed, low-protein diet, indoor system, outdoor system, subcutaneous adipose tissue, RNA-sequencing, nCounter gene expression assay

## Abstract

The Slovenian autochthonous breed, Krškopolje pig, is known for high fatness and better adaptability to different environmental conditions and feed resources. However, the metabolic processes underlying these adaptations, especially in response to different diets, have not yet been studied. A deeper understanding of these mechanisms could provide valuable insights into the breed’s adaptability to different environmental conditions. Therefore, the main objective of this study was to evaluate the effect of a low-protein (LP) diet on adipose tissue in Krškopolje pigs reared in either organic outdoor (n = 2 × 12) or conventional indoor (n = 2 × 14) systems. In the outdoor system, the LP diet had no effect on adipocyte size compared to the control (high-protein) diet, while it increased lipogenic enzyme activities and monounsaturated fatty acid content, and decreased polyunsaturated fatty acid content (*p* < 0.05). RNA sequencing revealed the upregulation of 28 genes and the downregulation of 37 genes. The upregulated genes were mainly involved in lipid metabolism (*ACLY*, *FASN*, *ACACA*, *MOGAT2*), oxidative stress, and mitochondrial function. In the indoor system, pigs on the LP diet had smaller adipocytes (*p* < 0.05), whereas no differences were detected in the lipogenic enzyme activities or fatty acid composition (*p* > 0.10). RNA sequencing revealed 30 upregulated and 28 downregulated genes. In the indoor system, heat shock proteins (*HSP70.2*, *HSPA6*) were upregulated in pigs on the LP diet, while genes involved in the innate immune system (*MSR1*, *TREM2*, *CSF3R*) were downregulated. To conclude, the present study showed that LP diet affected adipose tissue metabolism and gene expression in Krškopolje pigs, with different transcriptomic responses observed in outdoor and indoor rearing conditions.

## 1. Introduction

Local pig breeds can play an important role in sustainable pig production due to their lower nutritional demands, despite their lower growth performance and higher fat content. The Krškopolje pig, the only preserved autochthonous Slovenian pig breed, has not undergone genetic selection and consequently displays lower growth performance. Currently, Krškopolje pigs are raised using a wide variety of husbandry practices, ranging from intensive indoor farming to fully extensive outdoor systems. The majority are reared on small-scale farms, which usually use organic or extensive production methods [1]. Compared to modern, lean pig breeds, Krškopolje pigs exhibit greater fat deposition [2] and moderate protein deposition potential [3]. A recent study by Škrlep et al. [4] has shown that dietary protein deficiency has a greater impact on the performance of modern, lean breeds than fatty Krškopolje pigs. This indicates the adaptability of the breed, and lower dietary protein requirements. A pilot study by Brossard et al. [3] also showed that the nutrition of local pig breeds may not be adequately adapted to their needs. Several studies on other local pig breeds have investigated the effects of various diets on growth performance, carcass characteristics, and meat quality. In these studies, it was reported that a controlled reduction of the protein levels in the diet is a suitable strategy to influence intramuscular fat content and adipose tissue characteristics without significantly affecting growth [5,6,7,8,9]. Our recent research on Krškopolje pigs [10] showed that a protein-reduced diet affected carcass fatness only in the organic outdoor system, but not in the conventional indoor system, highlighting the interaction with the production system. Nevertheless, the mechanisms driving this underlying response, particularly in adipose tissue, are still poorly understood. Previous studies reported variable response of lysine-deficient or low-protein (LP) diets. For instance, research on Chinese local breeds [11,12] suggested an increase in lipogenic enzyme activity, while studies on Mediterranean breeds [5,6,13] found no notable effect on lipogenic enzymes or the gene expression of lipogenic genes. Given that LP diets may affect a broader range of biological processes beyond lipogenesis, including transcriptional regulation, it becomes important to explore their impact on the global gene expression landscape using RNA sequencing (RNA-seq). While the RNA-seq method is widely used in metabolic and nutrigenomic studies of modern pig breeds [9,14,15], it has never been used in local Krškopolje pig, highlighting the novelty and significance of the current study. Therefore, the main objective of this study was to elucidate how the adipose tissue transcriptomic profile responds to a reduced-protein diet in Krškopolje pigs raised in two different production systems: conventional indoor and organic outdoor systems.

## 2. Results

### 2.1. Histomorphological Traits of Adipose Tissue

In the indoor system, the LP diet was associated with smaller adipocyte size in the outer layer (*p* < 0.05) and showed a tendency toward smaller adipocytes in the inner layer (*p* < 0.10). Additionally, a tendency toward a higher adipocyte number per region of interest (ROI) was observed in the inner layer (*p* < 0.10). In the outdoor system, the LP diet group showed a tendency toward increased backfat thickness; however, no significant effect on adipocyte size or number was observed (Table 1).

### 2.2. Lipogenic Enzyme Activities of Adipose Tissue

The results of lipogenic enzyme activities primarily showed an effect of the diet on the adipose tissue in outdoor-reared pigs (Table 2). The LP diet significantly increased the activity of all four lipogenic enzymes tested in the subcutaneous adipose tissue (*p* < 0.05), with values ranging from 1.4- to over 2-fold differences. In contrast, no diet-related differences (*p* > 0.10) were observed in the indoor system.

### 2.3. Fatty Acid Composition of Adipose Tissue

The content of 20 different fatty acids was measured in the adipose tissue of Krškopolje pigs. The subcutaneous adipose tissue of animals fed the LP diet (Table 3) contained more monounsaturated fatty acids (MUFAs) (*p* < 0.05) and less polyunsaturated fatty acids (PUFAs) (*p* < 0.01) compared to those fed the control high-protein (HP) diet. This was particularly reflected in a higher proportion of oleic acid (C18:1) and a lower proportion of linoleic acid (C18:2n−6), together with some less representative PUFAs (e.g., C18:3n−3, C20:2n−6, C20:2n−3). In the indoor system, no notable differences were observed, except for alpha-linolenic (C18:3n−3, *p* = 0.001) and palmitoleic acid (16:1n−7, *p* < 0.10), both of which were lower in pigs fed the LP diet.

### 2.4. RNA-Sequencing

Sequencing yielded, on average, 66.8 million total reads. The average number of read counts was 69.0, 96.1, 51.7, and 49.7 million for the outdoor LP, outdoor HP, indoor LP, and indoor HP groups, respectively. Approximately 98.4% of the reads were mapped to the annotated *Sus scrofa* 11.1 genome, with 94.5% of the mapped clean reads having unique matches. General mapping statistics can be found in Supplementary File S1.

#### 2.4.1. Diet Effect on Differential Gene Expression in the Outdoor Rearing System

**Figure 1:**
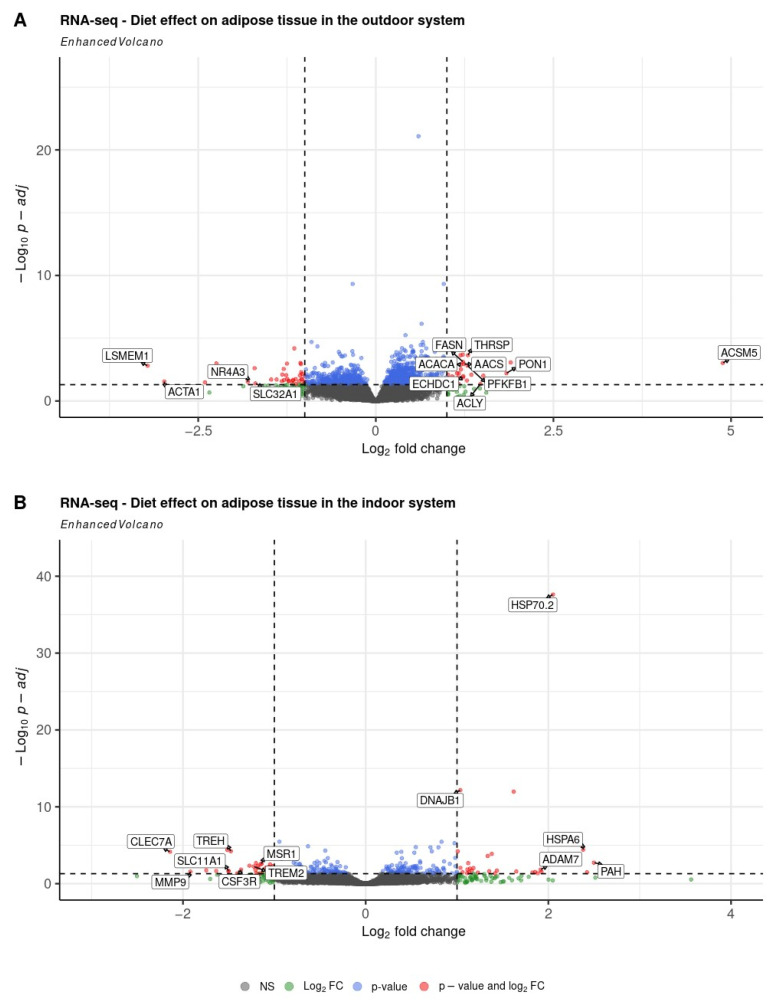
Volcano plots depicting genes expressed in the subcutaneous adipose tissue from pigs receiving low-protein (LP) vs. control high-protein diet (HP) in the (**A**) outdoor and (**B**) indoor systems. The horizontal lines indicate the significance threshold of differentially expressed genes at a *p*-value of 0.05. The vertical lines represent the threshold of |log_2_ (fold change)| > 1. Genes upregulated in the LP compared to HP group have log_2_(fold change) > 1, while downregulated genes have log_2_ (fold change) < −1.

**Figure 2:**
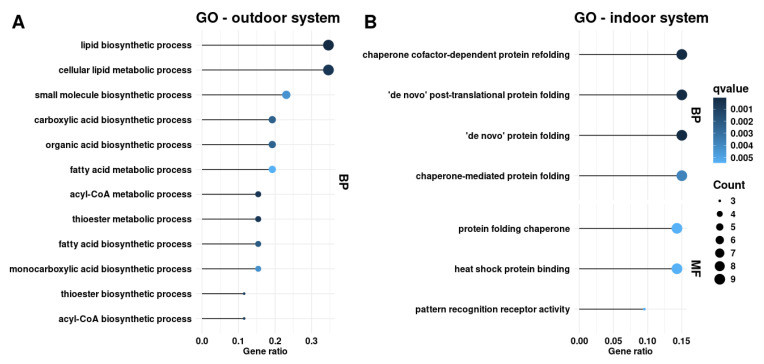
The gene ontology (GO) over-representation analysis of differentially expressed genes in the subcutaneous adipose tissue from pigs fed a low-protein (LP) versus control high-protein (HP) diet in the (**A**) outdoor and (**B**) indoor systems. GO analysis represents the biological process category (BP) and molecular function category (MF). The gene ratio refers to the proportion of differentially expressed genes relative to the total number of genes associated with a given GO term.

A comparison of LP and HP diets in the outdoor system revealed 65 differentially expressed genes (Figure 1A, Supplementary File S2). Among them, 28 genes were upregulated and 37 genes were downregulated, with log_2_ (fold change) values ranging from −3.2 (*LSMEM1*) to 4.9 (*ACSM5*). To annotate the differentially expressed genes, an over-representation analysis was performed, which revealed enrichment of genes involved in lipid metabolism (Figure 2A). These include terms related to lipid biosynthesis (GO:0008610), acyl-CoA metabolism (GO:0006637, GO:0071616), thioester metabolism (GO:0035383, GO:0035384), carboxylic acid (GO:0046394) and organic acid biosynthesis processes (GO:0016053) (Supplementary File S3). The KEGG over-representation analysis identified a significantly enriched pathway, namely propanoate metabolism (ssc00640) (Supplementary File S4).

#### 2.4.2. Diet Effect on Differential Gene Expression in the Indoor Rearing System

Differential gene expression analysis comparing LP and HP groups identified 58 differentially expressed genes (Figure 1B, Supplementary File S5). Among them, 30 genes were upregulated and 28 genes were downregulated with log_2_ (fold change) ranging from −2.1 (*CLEC7A*) to 2.5 (*PAH*). Gene ontology over-representation analysis revealed an enrichment of genes involved in biological processes and molecular functions related to chaperone cofactor-dependent protein folding (GO:0051085), ‘de novo’ post-translational protein folding (GO:0051084), and protein folding chaperone (GO:0044183) (Figure 2B, Supplementary File S6). KEGG over-representation analysis did not detect any significant pathways.

### 2.5. Candidate Gene Expression Analysis Using NanoString nCounter Gene Expression Assay

#### 2.5.1. Effect of Diet on the Candidate Gene Expression in the Outdoor System

**Figure 3:**
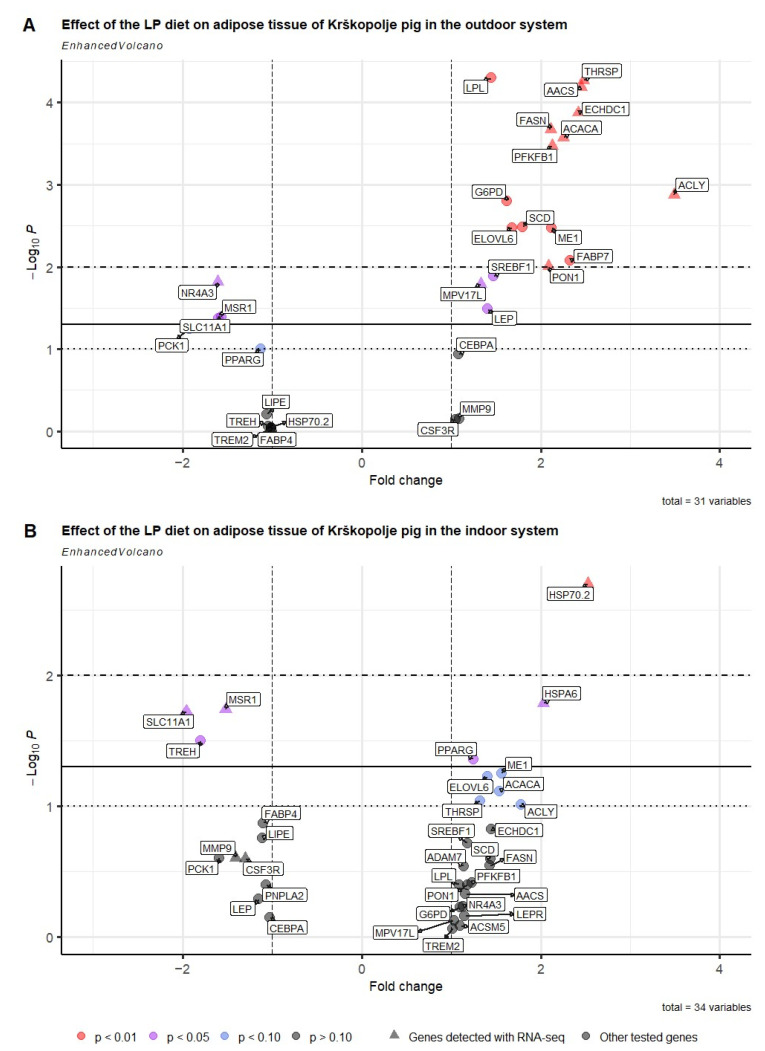
Volcano plot showing each gene’s −log_10_(*p*-value) and fold change in the (**A**) outdoor and (**B**) indoor systems comparing low-protein (LP) versus control high-protein (HP) diets in Krškopolje pig breed using the NanoString nCounter gene expression assay. The *x*-axis indicates fold change. Horizontal lines indicate *p*-value thresholds. Genes are colored in red if the resulting *p*-value is below 0.01, in purple if the resulting *p*-value is between 0.01 and 0.05, in blue if the resulting *p*-value is between 0.05 and 0.10, and in black if the resulting *p*-value is higher than 0.10. The triangles show genes that were also differentially expressed using the RNA sequencing method, while the circle denotes other tested genes. Genes upregulated in the LP compared to HP group have fold change > 1, while downregulated genes have fold change < −1.

In addition to the lipogenic genes already detected with RNA-seq (e.g., *FASN*, *ACACA*, and *ACLY*), the expression of several other candidate genes related to lipid metabolism was measured. In the LP group compared to the HP group (Figure 3A, Supplementary Files S7 and S8), *G6PD*, *ME1*, *SCD*, *ELOVL6*, and *LPL* genes (*p* < 0.05) were upregulated. On the other hand, *PCK1* and *PPARG* showed a tendency to be downregulated (*p* > 0.10) in LP group.

#### 2.5.2. Effect of Diet on the Candidate Gene Expression in the Indoor System

A comparison of the LP and HP groups (Figure 3B, Supplementary Files S7 and S8) showed that the *PPARG* gene was significantly upregulated (*p* < 0.05) in the LP group, while the expression of *ELOVL6*, *ME1*, *ACACA*, and *ACLY* tended to be increased (*p* > 0.10). Conversely, the expression of *TREH* was significantly downregulated in the LP group compared to the HP group (*p* < 0.05).

### 2.6. RNA-Seq Validation Using Nanostring nCounter Gene Expression Assay

To validate the RNA-seq results in the outdoor system, the expression of 10 genes was selected based on expression fold difference and due to the importance in lipid and energy metabolism (Figure 3A; Supplementary Files S7 and S8). The *MPV17L*, *PON1*, *FASN*, *ACACA*, *ECHDC1*, *AACS*, *THRSP*, *PFKFB1* and *ACLY* genes were upregulated (*p* < 0.01), while the *NR4A3* gene was downregulated in the LP compared to the HP group.

To validate the RNA-seq results in the indoor system, the expression of eight genes was measured (i.e., *ADAM7*, *CSF3R*, *HSPA6*, *HSP70.2*, *MMP9*, *MSR1*, *SLC11A1*, and *TREM1*) (Figure 3B, Supplementary Files S7 and S8). Among them, *HSPA70.2* and *HSPA6* were significantly upregulated (*p* < 0.05), while the *SLC11A1* and *MSR1* genes were downregulated (*p* < 0.05) in the LP compared to the HP group. Although the relative expression of the other four genes exhibited the same trend (i.e., *ADAM7*, *TREM2*, *MMP9*, and *CSF3R*), the difference was not significant (*p* > 0.10).

## 3. Discussion

### 3.1. The Effect of the Diet on Adipose Tissue of Krškopolje Pigs Reared in the Outdoor System

In the outdoor system, gene ontology enrichment analysis of RNA-seq data highlighted genes involved in lipid metabolism (*ACLY*, *FASN*, *ACACA*, *ACSM5*, *MOGAT2*, *ACSS2*, *RDH5*, and *CH25H*). Many of the upregulated genes in the LP compared to the HP group were directly related to fatty acid synthesis (*FASN*, *ACLY*, *ACACA*, *ACSM5*, and *THRSP*) or triglyceride synthesis (*MOGAT2*). This aligns with increased lipogenic enzyme activity and greater backfat thickness observed in the present study. Higher lipid deposition in pigs receiving LP diet could be due to lysine deficiency during fattening. In addition to higher *FASN* and *ACLY* gene expression, RNA-seq also revealed upregulation of the *ACACA* gene, which catalyzes the conversion of acetyl-CoA to malonyl-CoA. Malonyl-CoA is further catalyzed by *FASN* to synthesize palmitate. Both the *ACACA* and *FASN* genes have previously been identified as candidate genes for fatness and performance in pigs [15,16] and have been linked to fatty acid composition [17]. In the present study, upregulation of the *THRSP*, encoding thyroid hormone responsive spot 14, was detected, being a direct modulator of lipogenesis [18]. Polymorphisms and the expression of this gene have previously been associated with lipid synthesis and lipid concentration [19,20] and its expression has been linked to fatty acid composition [20]. The present study also detected the upregulation of the *MOGAT2* gene, which catalyzes the esterification of glycerol monoesters to diglycerides. This gene has previously been associated with larger fat deposition [21]. The targeted gene expression approach used in the present study additionally demonstrated the upregulation of *ME1* and *G6PD* genes, which provide nicotinamide adenine dinucleotide phosphate for fatty acid synthesis. Regarding the effects of the LP diet on lipogenic gene expression or enzyme activity, the relevant literature is generally very contradictory. For example, in fatty Wujin pigs [11], the LP diet led to increased expression of *ACACA*, *ME1*, and *G6PD* genes. On the contrary, the expression of the *ACACA* gene was downregulated in fatty Alentejano pigs receiving the LP diet [13]. In studies on the Iberian and modern breeds, no apparent effect of lysine-deficiency on lipogenic gene expression was detected [5,6]. In the present study, the targeted gene expression approach additionally revealed the upregulation of *SCD* and *ELOVL6*, genes responsible for the desaturation of SFA to MUFA. This is consistent with the higher MUFA content (especially oleic acid), and lower PUFA content in LP pigs reared outdoors. In contrast to our results, in a study on the Cinta Senese pig breed reared also outdoors [8], the MUFA content of subcutaneous adipose tissue remained unaffected by the LP diet, while lower SFA and higher PUFA levels were observed. These inconsistencies highlight the complexity of dietary effects on fat composition, which can be influenced by multiple factors. Variations in experimental conditions, such as the age of the animals, the extent of protein restriction, and environmental factors, likely contribute to the variability in reported results, making direct comparisons between studies challenging.

In the present study, RNA-seq analysis of the animals reared in the outdoor system additionally detected upregulation of genes involved in oxidative stress, such as paraoxonase 1 (*PON1*) and xanthine dehydrogenase (*XDH*), and mitochondrial functions (*MPV17L*, *ECHDC1*, *ACSM5*). Paraoxonase-1 is an antioxidant enzyme that maintains normal levels of lipid metabolism intermediates [22]. In addition, xanthine dehydrogenase is involved in adipocyte reactive oxygen species (ROS) generation under oxidative stress conditions [23]. ROS production is increased during adipocyte differentiation and the development of obesity [24], which also coincides with greater subcutaneous adipose tissue gain observed in LP pigs. The upregulation of genes involved in oxidative stress could also be a consequence of the environmental conditions, since animals in the outdoor system had increased energy demands for thermoregulation and physical activity [25,26]. Other upregulated genes (*ACSM5*, *ECHDC1*) detected in the present study were implicated in mitochondrial functions, more specifically with mitochondrial fatty acid β-oxidation [27]. Consistent with this, the transcriptional regulator *NR4A3*, which plays a role in lipid and carbohydrate metabolism including fatty acid β-oxidation, was downregulated in the LP group. Notably, studies have shown that knock-out of the *NR4A3* gene also impairs glucose uptake in adipocytes [28].

### 3.2. The Effect of the Diet on Adipose Tissue of Krškopolje Pigs Reared in the Indoor System

In the indoor system, Krškopolje pigs receiving the LP diet demonstrated enrichment of heat shock proteins (HSPs) (i.e., *HSP70.2*, *HSPA6*, and *DNAJB1*). HSPs are a family of proteins with protective functions under various stress conditions, such as nutritional stress, tissue hypoxia, or inflammatory response [29,30]. Elevated levels of intracellular HSP70 (i.e., *HSP70.2* and *HSPA6* genes) play a cytoprotective and anti-inflammatory role in adipose tissue, while extracellular *HSP70* exhibits proinflammatory activity when released into the extracellular space [31]. In the present study, the upregulation of HSPs in pigs fed the LP diet could be associated with anti-inflammatory and cytoprotective response, which is also supported by the downregulation of genes related to the innate immune system (*CLEC7A*, *TLR8*, *MSR1*, *TREM2*, *CPM*, *CSF3R*, and *SLC11A1*). Most of the immunity-related genes discovered in the present study are associated with macrophages, which are the most abundant immune cell type in adipose tissue and crucial for regulating adipose tissue homeostasis [32]. Macrophages perform a phagocytic function that involves the clearance of apoptotic and necrotic cells, and contributes to tissue remodeling [33]. For example, the LP diet in the present study affected the downregulation of the receptors *TREM2*, *MSR1*, and *CLEC7A*, which are expressed on immune cells (including macrophages) and act as receptors for different ligands [34,35,36]. In addition, a candidate gene expression approach used in this study showed the upregulation of the *PPARG* gene in the LP compared to HP group. This gene regulates adipogenesis, and supports the observed differences in histomorphological traits (i.e., a tendency toward a higher number of adipocytes per ROI in the inner layer) observed in LP compared to HP groups. Besides adipogenesis, *PPARG* can regulate key macrophage activities, such as differentiation, inflammation and polarization. The activation of *PPARG* has been reported to suppress the immune response of macrophages [33,37]. Accordingly, a downregulated metalloproteinase *MMP9* (encoding gelatinase B), which is involved in extracellular matrix degradation, was detected in the LP group by RNA-seq. *MMP9* can be expressed on fibrolytic macrophages, thus promoting phagocytosis (in HP compared to LP group) [32]. Furthermore, a study in mice has shown that LP diet decreases the number and functionality of macrophages [38] and that lysine deficiency can limit the synthesis of immunity-related proteins necessary for immune function [39]. The targeted candidate gene expression approach of the present study did not detect differences in the expression of genes involved in lipid metabolism or energy homeostasis. This aligns with the absence of phenotypic differences in adiposity-related traits (i.e., lipogenic enzyme activity and fatty acid composition), as well as no differences in backfat thickness. The most likely explanation is a much lower lysine content in the LP group of pigs raised indoors compared to those reared outdoors [10].

### 3.3. Conclusions

The present study on the local fatty Krškopolje pigs revealed that lowering dietary protein affected adipose tissue differently, depending on the environment. In the indoor system, the LP diet reduced the adipocyte size in the outer layer, while it did not affect lipogenic enzyme activities and fatty acid composition (measured in the inner layer). These findings were supported by both targeted and untargeted gene expression analyses, which showed no differences in lipid metabolism genes but indicated the suppression of genes involved in the immune system, particularly macrophages with phagocytic functions. In contrast, feeding an LP diet in the outdoor system resulted in increased lipogenic enzyme activities, higher MUFA, and lower PUFA content compared to the HP group. Gene expression analysis further confirmed the upregulation of genes associated with lipid deposition, fatty acid saturation, oxidative stress, and mitochondrial function.

## 4. Materials and Methods

### 4.1. Animals and Sample Collection

Two separate field trials were performed to test the effects of reduced dietary protein in a local pig breed. Krškopolje pigs were reared either in indoor (n = 2 × 14; 14 surgically castrated males and 14 females evenly distributed among the groups) or in outdoor conditions (fenced open area with trees and shelter) (n = 2 × 12; 16 surgically castrated males and 8 females evenly distributed among the groups). Animals from the outdoor system were reared according to organic standards and received organic feed ad libitum, while the animals reared in the indoor system received the same amount of feed based on the same ingredients, but of conventional origin. In both systems, animals received either standard (HP group: fed 15% crude protein from 20 to 80 kg, 12.5% crude protein from 80 to 100 kg, and 10% crude protein from 100 kg to slaughter) or reduced protein diet (LP group: fed 15% crude protein from 20 to 60 kg and 10% crude protein from 60 kg to slaughter). Diets were isoenergetic and isoproteic but differed in lysine content, with the organic diet containing less lysine. The trial began at an average age (mean ± SE) of 102 ± 6, 97 ± 4, 101 ± 2, and 103 ± 3 days for the outdoor HP, outdoor LP, indoor HP, and indoor LP groups, respectively. The corresponding initial body weights were 23.9 ± 0.8, 24.8 ± 0.7, 25.0 ± 1.1, and 24.9 ± 1.3 kg (mean ± SE). No significant differences were observed in the starting age or body weight between dietary regimes (*p*-values > 0.10). The animals were slaughtered at an average age of 331 ± 6, 326 ± 4, 329 ± 2, and 331 ± 3 days (mean ± SE), and body weights of 165.2 ± 4.5, 165.3 ± 2.7, 184.0 ± 3.9, and 182.5 ± 3.3 kg (mean ± SE) for the outdoor HP, outdoor LP, indoor HP, and indoor LP groups, respectively. No significant differences were observed in the final age or body weight within dietary regimens (*p*-values > 0.10).

Within the first 30 min after slaughter, carcasses were split apart and backfat thickness was measured at three anatomical sites (withers, last rib, and above *gluteus medius* muscle). The inner subcutaneous adipose tissue layer was collected at the level of the last rib and frozen in liquid nitrogen. The samples were stored at −80 °C for subsequent RNA extraction. For RNA-seq and NanoString nCounter gene expression assay, 24 subcutaneous adipose tissue samples from the indoor group (n = 2 × 12) were selected, along with all samples from the outdoor system group (n = 2 × 12). For the determination of histomorphological traits, lipogenic enzyme activities, and fatty acid composition, the subcutaneous adipose tissue of all animals was collected (n = 2 × 14 for the indoor and 2 × 12 for the outdoor comparisons).

### 4.2. Adipose Tissue Histomorphological Traits

The cellularity of adipose tissue was measured as described in Škrlep et al. [4]. Briefly, the inner and the outer subcutaneous adipose tissue layers sampled at the level of the last rib (a cuboid of 2 × 1 × 1 cm) were dissected and fixed in 10% buffered formalin. Tissue sections of 5 µm (microtome Leica SM2000R, Nussloch, Germany) were cut in a vertical plane through the dermis and both layers of subcutaneous adipose tissue. The sections were stained with hematoxylin and eosin (HE) and cover slipped (Gemini AS slide stainer and cover slipper ClearVue, Thermo Fisher Scientific, Runcorn, UK). For histomorphometric analysis, images were obtained by a light microscope (Eclipse Ni-U, Nikon, Tokyo, Japan) and a digital camera (DS -Fi1, Nikon, Tokyo, Japan). For measuring the histomorphological parameters of the inner and outer subcutaneous adipose tissue layer, 3 images per sample (each measuring 1.1 mm^2^) were obtained for each layer at 10× objective magnification, and the number and cross-sectional area (CSA) of adipocytes were determined using macro protocol in Fiji 1.53 software [40].

### 4.3. Determination of Lipogenic Enzyme Activity

Fatty acid synthase (FAS), citrate cleavage enzyme (CCL), glucose-6-phosphate dehydrogenase (G6PD), and malic enzyme (ME) were determined in subcutaneous adipose tissue according to the original methods of Bazin and Ferré [41]. Approximately 1 g of frozen pulverized sample was homogenized in 10 mL of ice-cold sucrose buffer (0.25 M sucrose, 1 mM ethylenedinitrilotetraacetic acid, 1 mM dithiothreitol, and 1 mM phenylmethylsulphonyl fluoride) and the homogenate centrifuged at 12,000× *g* at 0 °C for 1 h. The activities of lipogenic enzymes were assessed spectrophotometrically by measuring the absorbance at 340 nm against the blank for 10 min at 37 °C.

### 4.4. Fatty Acid Composition Analysis

Fatty acid composition of subcutaneous adipose tissue was assessed as previously described in Škrlep et al. [42]. After pulverizing the samples in liquid nitrogen, 100 mg of subcutaneous adipose tissue were weighed and transmethylated in situ in 0.5 M NaOH solution in methanol and dichloromethane (incubated for 50 min at 90 °C) and afterwards by the addition of 14% BF3 in methanol (incubated for 10 min at 90 °C). The fatty acid methyl esters (FAMEs), resulting from the reaction, were extracted using hexane. The separation of FAMEs was performed by Hewlett–Packard 6890 Gas Cromatograph (Agilent, Santa Clara, CA, USA) fitted with Supelco SP-2560 Capillary column (100 m × 0.25 mm i.d. × 0.20 μm; Supelco, Bellafonte, PA, USA) using nitrogen as a carrying gas and flame-ionization detector (FID). The initial temperature set at 80 °C was increased in the following order: to 160 °C at 20 °C/min, then to 198 °C at 1 °C/min for a total running time of 94.4 min. The temperature of the injector was set at 220 °C and of the FID to 200 °C. Specific FAMEs were identified using mixtures of standards (Supelco 37 Component FAME mix), while nonadecanoic acid was used as an internal standard to determine tissue fatty acid concentrations.

### 4.5. RNA-Sequencing Analysis

#### 4.5.1. RNA Extraction, Library Preparation, and Sequencing

Total RNA was extracted from approximately 90 mg of subcutaneous adipose tissue using the RNeasy Lipid Tissue Mini Kit (Qiagen GmbH, Hilden, Germany) according to the manufacturer’s instructions. The quality of the extracted RNA was assessed by measuring the ratio in optical density between 260 and 280 nm using Eppendorf BioSpectrometer (Eppendorf AG, Hamburg, Germany). Samples were quantified using Qubit 4.0 Fluorometer (Life Technologies, Carlsband, CA, USA). The integrity of RNA was assessed with Agilent 2100 Bioanalyzer (Agilent, Santa Clara, CA, USA). The extracted RNA samples had an RNA integrity number larger than 6.2 and OD260/OD280 ratio between 1.7 and 2.0. Prior to library preparation, equal amounts of total RNA from two individuals were pooled together, generating 6 pools within each dietary group.

RNA sequencing libraries were prepared using the NEBNext Ultra II RNA Library Prep Kit for Illumina (NEB, Ipswich, MA, USA) according to the manufacturer’s instructions. The libraries were validated using an NGS Kit on an Agilent 5300 Fragment Analyzer (Agilent Technologies, Palo Alto, CA, USA) and quantified by a Qubit 4.0 Fluorometer (Life Technologies, Carlsband, CA, USA).

The multiplexed sequencing libraries were loaded onto the Illumina NovaSeq 6000 generating 2 × 150 paired-end reads. Raw sequence data were converted into fastq files and de-multiplexed using Illumina bcl2fastq program (version 2.20). The RNA-seq experiment was submitted to the NCBI Gene Expression Omnibus (GEO) (Bethseda, MD, USA) and was assigned GEO accession number GSE292746.

#### 4.5.2. Bioinformatic Analysis of RNA-Seq Data

The quality control of the raw data was performed using FastQC v1.0.0 software. The sequence reads were trimmed using Trimmomatic v.0.36 [43] to remove adapter sequences and poor-quality nucleotides. The trimmed reads were mapped to *Sus scrofa* 11.1 reference genome (release 108) using STAR aligner v.2.5.2b [44]. Unique gene hit counts were calculated using featureCounts (Subread package v.1.5.2.) [45] and summarized using the gene_id feature in the annotation file. Unique reads that fell within the exon region were counted.

Gene hit counts were further used for differential gene expression analysis using DESeq2 [46]. In both production systems (i.e., outdoor and indoor systems), differentially expressed genes in the LP compared to HP diets were searched. The Wald test was used to generate and calculate *p*-values and log_2_ fold changes. The resulting *p*-values were adjusted using the Benjamini–Hochberg method. Significant differential expression threshold was set at the adjusted *p*-value of 0.05 and |log_2_(fold change)| > 1. Volcano plots were created using EnhancedVolcano R package.

#### 4.5.3. Gene Ontology and KEGG Pathway Over-Representation Analyses

Gene ontology (GO) over-representation analysis of differentially expressed genes was performed by the R package ClusterProfiler v.3.17 [47]. Gene ontology terms with the adjusted q value (adjusted *p*-value by Benjamini and Hochberg method) below 0.05 were considered significantly enriched.

Additionally, R package ClusterProfiler v.3.17 [48] was used to test the statistical enrichment of differentially expressed genes in the Kyoto Encyclopedia of Genes and Genomes (KEGG) pathways using Fisher’s exact test. Test results were subjected to multiple testing corrections of the *p*-values by the Benjamini and Hochberg method.

### 4.6. Candidate Gene Expression Analysis and Validation of RNA-Seq Data Using NanoString

A total of 48 test genes with 5 housekeeping genes were selected for gene expression quantification in individual animals on the nCounter Sprint (NanoString Technologies, Seattle, WA, USA) using Elements TagSets. Test genes were selected from the list of differentially expressed genes detected by RNA-seq based on expression fold difference and due to their importance in lipid and energy metabolism. Additional genes were selected from the literature survey.

For the outdoor group, the following candidate genes were selected from the literature: *CEBPA*, *ELOVL6*, *G6PD*, *LEP*, *LIPE*, *LPL*, *ME1*, *PCK1*, *PNPLA2*, *PPARG*, and *SCD*. For RNA-seq validation, the following genes were selected from the list of differentially expressed genes: *AACS*, *ACACA*, *ACLY*, *ACSM5*, *ECHDC1*, *FASN*, *MPV17L*, *PFKFB1*, *PON1*, *THRSP*, *NR4A3*, *MOGAT2*, *TRPV4*, and *XDH*. For the indoor group, the following candidate genes were selected from the literature: *ACACA*, *ACLY*, *CEBPA*, *ELOVL6*, *FABP4*, *FASN*, *G6PD*, *LEP*, *LEPR*, *LIPE*, *LPL*, *ME1*, *PCK1*, *PNPLA2*, *PPARG*, *SCD*, *SREBF1*, and *TREH*. For RNA-seq validation, the following genes were selected: *CSF3R*, *HSP70.2*, *MMP9*, *MSR1*, *SLC11A1*, *TREM2*, *ADAM7*, *ATP6V0D2*, *CLEC7*, *SLC27A5*, and *TAS2R40*. More details are provided in Supplementary File S8.

The oligonucleotide probes used were designed by NanoString Technologies (Seattle, WA, USA) and synthesized by IDT Technologies (Coralville, IA, USA) (Supplementary File S8). Prior to analysis, RNA samples were diluted to 40 ng/μL in RNAse-free water. The assay was performed according to the manufacturer’s protocol.

The nSolver analysis software v.4.0 was used for the raw data quality control (i.e., reporter code count files or RCC files) analysis. The RCC files were imported and underwent software quality control routine. For all samples, fields of view registration (imaging) was more than 99%; binding density was between 0.2 and 0.71; positive control linearity was 1.0; and the limit of detection was more than 0.5 fM positive control and ≤2 standard deviations above the mean of the negative controls.

The background threshold count value was defined as the average value plus two standard deviations of negative control probes. Testing genes having more than 40% of samples above background threshold count value were further considered for the analysis. Counts of eight genes (*MOGAT2*, *TAS2R40*, *TRPV4*, *XDH*, *SLC27A5*, *ATP6V0D2*, *CLEC7A*, and *PAH*) were below threshold; therefore, they were excluded from the analysis. Gene *FABP7* was excluded from the indoor comparison, and genes *LEPR*, *ACSM5*, *HSPA6*, and *ADAM7* were excluded from the outdoor comparison due to a low number of samples being above background threshold (Supplementary Files S8). Positive control normalization factor was computed using geometric mean. Afterwards, the housekeeping normalization factor was calculated using geometric mean of 4 housekeeping genes, i.e., *B2M*, *PPIA*, *TOP2B*, and *YWHAZ* with the percentage of coefficient of variation ranging from 13.6 to 18.9% for the indoor comparison, and 22.5 to 26.7% for the outdoor comparison. Housekeeping gene *GAPDH* was excluded from the normalization due to a high coefficient of variation. A Welch *t*-test was performed to calculate the significance of the fold change. EnhancedVolcano R package was used to present the results.

### 4.7. Statistical Analysis

Data from histomorphological analyses, lipogenic enzyme activities, and fatty acid composition were analyzed using SAS statistical software version 9.1 (SAS Institute Inc., Cary, NC, USA) with the PROC MIXED procedure. The effect of diet was evaluated within conventional indoor and organic outdoor rearing systems. In both cases, the model included fixed effects of the diet and sex. Least square means were calculated and compared using the LSMEANS procedure with the PDIFF option.

## Figures and Tables

**Table 1:** Histomorphological characteristics of subcutaneous adipose tissue in Krškopolje pigs according to feeding in outdoor and indoor rearing systems.

	HP Diet	LP Diet	RMSE	*p*-Value
**Outdoor rearing system**				
Number of pigs	12	12		
Backfat thickness, mm	49.4	53.7	5.21	0.058
Adipocyte surface area, µm^2^				
Inner layer	7828	8055	873.1	0.540
Outer layer	8596	8645	723.1	0.872
Number of adipocytes per ROI				
Inner layer	136	132	24.0	0.686
Outer layer	149	144	24.3	0.622
**Indoor rearing system**				
Number of pigs	14	14		
Backfat thickness, mm	45.1	42.7	5.24	0.236
Adipocyte surface area, µm^2^				
Inner layer	7330	6767	855.5	0.094
Outer layer	8048	7321	792.1	0.023
Number of adipocytes per ROI				
Inner layer	160	180	28.8	0.079
Outer layer	185	190	23.7	0.548

HP = control high-protein diet; LP = low-protein diet; RMSE = root mean square error; ROI = region of interest.

**Table 2:** Lipogenic enzyme activities in subcutaneous adipose tissue of Krškopolje pigs according to the dietary treatment in outdoor and indoor rearing systems.

	HP Diet	LP Diet	RMSE	*p*-Value
**Outdoor rearing system**				
Number of pigs	12	12		
ME1	298.5	416.4	108.24	0.014
G6PD	500.7	628.4	127.69	0.023
CCL	167.4	256.0	71.3	0.006
FASN	19.5	39.5	19.8	0.034
**Indoor rearing system**				
Number of pigs	14	14		
ME1	211.6	240.8	60.27	0.212
G6PD	491.3	495.1	102.7	0.922
CCL	123.9	124.1	53.59	0.992
FASN	18.1	15.0	8.77	0.456

HP = control high-protein diet; LP = low-protein diet; RMSE = root mean square error; ME1 = malic enzyme; G6PD = glucose-6-phosphate dehydrogenase; CCL = citrate cleavage enzyme; FASN = fatty acid synthase. The activities of ME1 and G6PD are expressed as nmol of reduced nicotinamide adenine dinucleotide phosphate per minute per gram of wet tissue, and the activities of CCL and FASN are expressed as nmol of oxidized nicotinamide adenine dinucleotide phosphate per minute per gram of wet tissue.

**Table 3:** The fatty acid composition of subcutaneous adipose tissue of Krškopolje pigs according to the dietary treatment in outdoor and indoor rearing systems. Results are in g/100 g of fatty acids.

	HP Diet	LP Diet	RMSE	*p*-Value
**Outdoor rearing system**				
Number of pigs	12	12		
SFA	43.47	43.52	1.570	0.937
MUFA	45.55	46.73	1.273	0.034
PUFA	10.98	9.75	0.859	0.002
C10:00	0.05	0.05	0.016	0.787
C12:00	0.07	0.07	0.004	0.606
C14:00	1.36	1.31	0.060	0.066
C15:00	0.01	0.00	0.010	0.204
C16:00	25.79	25.93	0.724	0.637
C16:1n−7	1.8	1.86	0.267	0.616
C17:00	0.13	0.12	0.014	0.015
C17:1n−7	0.21	0.19	0.031	0.080
C18:00	15.83	15.8	1.248	0.950
C18:1n−9	42.18	43.26	1.144	0.031
C18:2n−6	9.26	8.21	0.757	0.003
C18:3n−3	0.69	0.61	0.058	0.005
C20:00	0.24	0.25	0.020	0.194
C20:1n−9	1.36	1.43	0.150	0.275
C20:2n−6	0.69	0.62	0.058	0.009
C20:3n−6	0.05	0.04	0.008	0.061
C20:3n−3	0.13	0.11	0.013	0.006
C22:1n−9	0.00	0.00	.	.
C20:4n−6	0.17	0.15	0.023	0.186
C22:6n−3	0.00	0.00	.	.
**Indoor rearing system**				
Number of pigs	14	14		
SFA	41.71	42.15	1.617	0.483
MUFA	46.52	46.82	1.661	0.342
PUFA	11.77	11.03	1.145	0.103
C10:00	0.06	0.06	0.005	0.178
C12:00	0.07	0.07	0.007	1.000
C14:00	1.30	1.26	0.080	0.130
C15:00	0.03	0.02	0.022	0.239
C16:00	25.01	24.79	0.806	0.465
C16:1n−7	2.01	1.83	0.246	0.059
C17:00	0.14	0.14	0.022	0.768
C17:1n−7	0.25	0.25	0.038	0.518
C18:00	14.89	15.60	1.181	0.123
C18:1n−9	43.09	43.48	1.533	0.509
C18:2n−6	9.99	9.36	0.994	0.108
C18:3n−3	0.84	0.73	0.076	0.001
C20:00	0.21	0.22	0.027	0.453
C20:1n−9	1.17	1.24	0.952	0.292
C20:2n−6	0.60	0.61	0.065	0.804
C20:3n−6	0.04	0.04	0.008	0.749
C20:3n−3	0.11	0.10	0.024	0.235
C22:1n−9	0.00	0.01	0.038	0.327
C20:4n−6	0.18	0.19	0.027	0.810

HP = control high-protein diet; LP = low-protein diet; RMSE = root mean square error; SFA = saturated fatty acid; MUFA = monounsaturated fatty acid; PUFA = polyunsaturated fatty acid.

## Data Availability

RNA-sequencing data are available under accession GSE292746. Other data are available upon direct request to the authors.

## Supplementary Materials

The following supporting information can be downloaded at: https://www.mdpi.com/article/10.3390/ijms26094440/s1.
